# Draining Fluids through a Peritoneal Catheter in Newborns after Cardiac Surgery Helps to Control Fluid Balance

**DOI:** 10.1155/2010/731865

**Published:** 2010-03-30

**Authors:** Elisa Ruano Cea, Philippe Jouvet, Suzanne Vobecky, Aicha Merouani

**Affiliations:** ^1^Department of Pediatric Nephrology, CHU Sainte-Justine Hospital, University of Montréal, 3175, Côte Sainte Catherine, Montréal, QC, Canada H3T 1C5; ^2^Pediatric Intensive Care, Sainte-Justine Hospital, University of Montréal, QC, Canada H3T 1C5; ^3^Cardiac Surgery, Sainte-Justine Hospital, University of Montréal, QC, Canada H3T 1C5

## Abstract

Fluid overload, a common complication following cardiac surgery in infants is often difficult to manage. Dialysis can be used in severe cases, but may not be well tolerated. In such patients, peritoneal drainage could be an alternative option for fluid removal. We report the case of a newborn with a truncus arteriosus who developed postoperatively a complicated clinical course with right ventricular dysfunction, prerenal condition as well as fluid overload despite diuretic therapy. Dialysis was indicated for fluid removal. Peritoneal dialysis was started using a surgically placed Tenckhoff catheter and stopped due to inefficacy and leaks and no other modalities of dialysis were used. However, the catheter was left in place over a period of two months for fluid drainage and removed because of unexplained fever. In order to determine the effect of peritoneal drainage, we selected a period of one week before and one week after the removal of the drain to compare daily clinical data, urine electrolytes and renal function and found a positive effect on fluid balance control. We conclude that the fluid removal by continuous peritoneal drainage is a simple and safe alternative that can be used to control fluid balance in infants after cardiac surgery.

## 1. Introduction

Fluid overload is a common complication following cardiac surgery in infants. The cause is thought to be multifactorial and includes pre-existing cardiac dysfunction, exogenous fluid during cardiopulmonary bypass, postsurgery acute kidney injury and the strain on the heart caused by the surgical procedure [1–3]. Conservative management such as the use of diuretics is often insufficient to adequately manage fluid overload. Ultrafiltration through peritoneal dialysis or hemodialysis can be used in severe cases [4, 5], but may not be well tolerated. The insertion of a peritoneal drain and its use to remove peritoneal fluid postoperatively is commonly used in pediatric cardiac surgery especially when newborns with complex diseases are involved but data have never been published. In this case report, we describe a newborn patient in whom a peritoneal drain helped the management of his fluid balance after cardiac surgery.

## 2. Case Report

The patient described here was born to a mother known with insulin dependant type 2 diabetes. The pregnancy was overall uncomplicated, but antenatal ultrasounds revealed the presence of a truncus arteriosus. Delivery was induced at 40 weeks gestational age and was complicated by shoulder dystocia as well as thick meconium fluid, but no invasive resuscitation was required. Birth weight was 4.0 kg and Apgars were 7, 9, 9 at 1, 5 and 10 minutes, respectively.

Cardiac ultrasound confirmed the presence of only congenital abnormalities with a truncus arteriosus type I associated with atrial and ventricular septal defect. Preoperative renal function evaluation was normal with serum creatinine of 30 *μ*mol/L, blood urea nitrogen of 2.2 mmol/L; urine analysis was normal as well as renal Doppler ultrasound. Preoperative weight was 3.9 kg. The corrective cardiac surgery was performed at 8 days of life. During extracorporeal circulation that lasted 198 minutes, a modified ultrafiltration used during 36 minutes drained 275 milliliters of fluid. The aortic cross was clamped during 60 minutes. At the end of the surgery, fluid balance was positive of 550 cc. During the follow-up, the patient had multiples episodes of worsening renal function and fluid retention despite diuretic therapy. It was a prerenal dysfunction with a fractional excretion of sodium of less than 1% consistent with a hemodynamic cause. The first episode of renal dysfunction began immediately after cardiac surgery. Serum creatinine concentration increased from a preoperative value of 30 *μ*mol/L to 107 *μ*mol/L on day 1 with a maximum of 120 *μ*mol/L on day 4 and returned to normal with value of 30 *μ*mol/L on day 11 after surgery. In parallel, the cardiac function remained difficult to stabilize after the surgery with a decrease in the contractility of the right ventricle noted on day 9 postsurgery. Three weeks postsurgery, the patient had remained on ventilatory support because of persistent cardiac failure mainly due to right ventricular dysfunction. On post-operative day 23, the serum creatinine concentration reached a peak of 105 *μ*mol/L, and the patient's weight was 60% over his birth weight. Low volume peritoneal dialysis through a surgically placed cuffed Tenckhoff catheter was initiated with 10 mL/kg exchange volume and the dwell time was one hour. Glucose-based peritoneal dialysis solutions were used with concentration of glucose of 1.5% and 2.5%. After repetitive episodes of blood pressure drop following the drainage with incidental hypoxemia and difficulties with dialysate leaks, peritoneal dialysis had to be discontinued 3 days later. The peritoneal drain was kept in place and used for continuous drainage over a period of 2 months from day 23 to day 85 in an attempt to prevent or treat significant fluid accumulation and was removed because of an unexplained fever despite multiples investigations. Over this period, there were no signs of infection at entry site or peritonitis and peritoneal fluid cultures remained negative.

In order to determine the effect of peritoneal drainage on fluid balance and diuresis, we analyzed the data from the week prior and following the discontinuation of the peritoneal drainage on day 85 (Table 1). During this period, the patient remained stable as shown by similar use of inotropic medication (milrinone, digoxin, clonidine) and PEdiatric LOgistic Dysfunction score (PELOD) [6]. The daily dose of furosemide medication was also similar over the 2 weeks period (4.5 mg ± 0 and 4.5 ± 1.5 mg/kg/day used as boluses). Diuresis remained unchanged throughout the period analyzed (2.6 ± 0.7 cc/kg/hour and 3.1 ± 0.5 cc/kg/hour); yet the median daily in and out fluid balance increased from +53 mL/day (range: −309 to +174) with the peritoneal drain to +131 mL/day (range −150 to +339) after the removal of the drain; the median drained fluid volume was 1 cc/kg/hour (range: 0.3–2.3). The urinary sodium concentration remained below 10 mmol/L with fractional excretion of sodium of less than 1%, and serum creatinine level was comparable over the two weeks period (40 ± 6 and 45 ± 9 *μ*mol/L). Although peritoneal drainage slightly improved the fluid balance, the clinical course of the patient worsened; he remained ventilated because of both cardiac and thoracic oedema and died on day 118 after surgery from decompensated cardiac failure, sepsis, pulmonary infection, and worsening renal function.

## 3. Discussion

We present the case of an infant in whom a continuous peritoneal drain was used successfully to assist the management of fluid overload after cardiac surgery without decreasing urine output nor impairing renal function. Fluid overload is a common complication following cardiac surgery in infants. Several treatments have been proposed to treat fluid overload in the context of cardiac failure and cardiac surgery including diuretics and peritoneal dialysis [1–5]. Most patients with heart failure who require diuretic therapy are treated with loop diuretics as they are powerful agents [7]. However, despite high dose of furosemide up to 5 mg/kg/day that were used in this patient, there was a low responsiveness to this medication as shown by a persistently low urine ratio of Na/K < 1, a fractional sodium excretion of less than 1% and urine output not exceeding 3 mL/kg/day. In cases of acute kidney injury and persistent fluid overload refractory to diuretic therapy, peritoneal dialysis is a safe and adequate treatment. However, complications such as leaks, ultrafiltration failure in patient with hemodynamic instability, abdominal distension, hyperglycemia induced by osmotic dialysate and pulmonary complications may limit its indications [8]. Because our patient developed repetitive episodes of blood pressure drop following the drainage and technical difficulties with dialysate leaks, and the need for dialysis was not necessary as renal function improved, it was decided to stop the dialysis and keep the Tenckoff catheter for peritoneal drainage. The use of peritoneal drainage for the management of fluid overload following cardiac surgery in newborns is commonly used in practice but data have not been published. One study had reported a positive impact on fluid balance in the treatment of patient with ascitis [9]. In the present case, when comparing the week preceding and following the removal of the peritoneal drain, we noted that the urine output was similar, but that the fluid balance could only be improved with the help of the peritoneal drain. During the study period, the daily dose of furosemide remained similar, while spirolonactone was discontinued over the second period because of its weak diuretic effect [10] and its inability to avoid furosemide induced hypokalemia. In addition, fluid removal by peritoneal drainage did not impair renal function.

## 4. Conclusion

Cardiac surgery in the newborn is often complicated by fluid overload. While peritoneal dialysis is needed to help regulate fluid balance when acute kidney injury is present, the continuous peritoneal drainage is a simple alternative technique to remove fluid in these patients when dialysis is not indicated. Further data collection from other similar patients is however needed to confirm this hypothesis.

## Figures and Tables

**Table 1:** Effect of the peritoneal drain on fluid balance after cardiac surgery. During the week preceding and following peritoneal drain removal we report the clinical severity data (PELOD score), diuretic therapy, parameters of the fluid balance, and renal function. Data were compared with the bilateral student *T* test. *P*-value <  .05 was considered statistically significant.

	Peritoneal drain	No peritoneal drain	*P* value
PELOD score	8 ± 5	7 ± 5	.63
Furosemide (mg/kg/day)	4.5 ± 0	4.5 ± 1.5	.50
In/out Fluid balance (mL/day)	+53 (−309, +174)	+131 (−150, +339)	.06
Urine output (mL/kg/hr)	2.6 ± 0.7	3.1 ± 0.5	.19
Drain output (mL/kg/hr)	1 (0.3, 2.3)	0	
FENa*	<10	<10	
Serum creatinine (*μ*mol/L)	40 ± 6	45 ± 9	.22

*FENa: fractional excretion of sodium. (Results are expressed as median and range for fluid balance, drain and urine output and as mean and SD for Pelod score, furosemide therapy and renal function).
